# Fœtal Auscultation

**Published:** 1874-09

**Authors:** Albert B. Strong

**Affiliations:** Ex-Resident Physician Cook County Hospital, Chicago; 312 West Indiana Street


ARTICLE V.—*Fœtal Auscultation—Report of Fifty Cases.* By ALBERT B. STRONG, M.D., Ex-Resident Physician Cook County Hospital, Chicago. Read before the Cook County Medical Society, July 20, 1874.

It is claimed that the pulse of the female fœtus is uniformly much faster than that of the male, and consequently it is possible to determine the sex of the child in utero. According to one observer, there is a difference of fifteen or twenty pulsations per minute. It is also claimed, that the presentation may be accurately diagnosed by noting the point at which the heart-sounds are heard the plainest.

For the purpose particularly of investigating the truth contained in these statements, the following observations were performed upon patients in the Lying-in department of Cook County Hospital of this city.

During the examination, which embraced fifty cases, many interesting facts were elicited which will first be laid before the Society. As the heart-sounds are usually limited to a small space of two or three inches square, the stethoscope will be found to be preferable to the unaided ear.

Aside from the rumbling in the intestines, two distinct sounds may be heard, first, the utero-placental souffle which is syncronous with the maternal pulse. This is a loud, blowing murmur, frequently of a musical character, and is not at all constant, being plainly audible at one moment and absent the next. It differs very materially from the heart-sounds which are independent of the maternal circulation, and is short, sharp, clear and clicking, similar in character to the second sound of the adult heart.

The fœtal pulse is subjected to the same variations as that of the adult, only in a much more marked degree. While the child is perfectly quiet, the heart is tolerably uniform in its pulsations; but let it make the least movement and almost instantly the pulse is greatly accelerated, increasing thirty or forty beats in the minute. The cause being removed, it falls as rapidly as it ascended. Though the child be alive, it is said the heart-sounds cannot always be heard. Such cases must be rare, especially in the last month of pregnancy, for we never have failed, after a careful, and in some cases prolonged, examination, in detecting it. Frequently at the commencement of the observation, the souffle has been so intense as to mask all other sounds, but as the momentary excitement of the mother passed off, the heart could be heard. Throughout a natural labor the action of the heart is variable, so much so that in the majority of the cases where the observation was taken at this time alone, the diagnosis proved to be wrong. For the first part of a pain, the pulse is greatly accelerated, but as the uterus contracts more firmly, and the child is subjected to greater pressure, it falls considerably; as the pain passes off, it rises above its natural pulsation, and, if the interval last a few moments, it again falls and becomes regular at a more frequent pulsation than before labor began. As, for example, in one case the pulse was steady for several days at 124, in the first part of a pain it ran up to 150, at the latter part it sank to 60; in the first part of the interval it increased to 170, and then decreased, and became steady at 134.

From these facts in regard to the variable action of the fœtal heart, it is evident that the examination *must be repeated at different intervals and during the time that the child is perfectly quiet*, and that the average result of such examinations, being an approximation to the true state of the pulse, can alone be of possible service in determining the sex of the child. In searching the literature of the subject, which is exceedingly scant, we have been much surprised to see how other observers have arrived at conclusions so radically different from our own.

In an article on Fœtal Physical Diagnosis, (published in the *American Practitioner*, of December, 1873,) by Frank C. Wilson, M.D., Visiting Physician to the Louisville City Hospital, the Doctor says he has kept "accurate notes of all the cases met with in hospital and private practice, diagnosing the sex in each, failing only in nine cases out of one hundred and nine." 125 was the average pulse of the males, 143 that of the females, 134 being the average of both sexes, and the dividing line between them. For Dr. Cumming's 41 cases the average pulse of the males was 131; of the females, 145; of both sexes, 136. For our own cases there is not this great difference between the pulse of the male and female, and consequently the ability to predict the sex is far from flattering.

The record as kept at the bedside, is herewith submitted, in order that those who are so disposed may draw their own conclusions. This privilege is denied in the article above alluded to. The weight of most of the children was also taken, under the supposition that there might be some relation between the rapidity of the pulse and the vigor of the child as indicated by this; no connection, however, is found.

For convenience, the uterus was divided by right lines, into four equal parts, designated as the right and left upper, and the right and left lower quarters.

It is said, if the heart pulsates in the lower half of the abdomen, the vertex presents; if in the upper, the breech is the presenting part; and furthermore, that the occiput or coccyx will be on that side of the median line where the heart-sounds are heard the plainest.

The table alluded to is as follows :

| No. of Patient. | Date of Observation. | Pulsation of Heart. | Where Heard. | Date of Delivery. | Presentation. | Position. | Sex. | Weight. lbs. |
|---|---|---|---|---|---|---|---|---|
| 1 | Sept. 18th, 1873 | 124 | Left lower quarter. | Sept. 30th. | Ver. | 1st. | M. | |
| 1 | 2nd stage labor | 132 | " " | | | | | |
| 2 | Sept. 26th | 128 | " " | Oct. 1st. | Ver. | 1st. | F. | |
| 3 | Sept. 16th | 132 | Right lower quarter. | Oct. 4th. | Ver. | 2d. | F. | |
| 3 | Sept. 22nd | 140 | " " | | | | | |
| 3 | Oct. 1st | 140 | " " | | | | | |
| 4 | Oct. 1st | 140 | " " | Oct. 1st. | Ver. | 2d. | M. | |
| 5 | Oct. 11th | 128 | Left lower quarter. | Oct. 11th. | Ver. | 2d. | M. | 9 |
| 6 | Oct. 6th | 124 | Right lower quarter. | Oct. 11th. | Ver. | 1st. | F. | 7½ |
| 7 | Oct. 8th | 130 | Left lower quarter. | Oct. 16th. | Ver. | 1st. | F. | 8½ |
| 7 | 1st stage labor | 140 | | | | | | |
| 7 | 2nd stage labor | 144 | | | | | | |
| 8 | Oct. 9th | 154 | " " | Oct. 16th. | Ver. | 1st. | F. | 9 |
| 9 | 2nd stage labor | 140 to 160 | Median line between pubes and umbilicus. | Oct. 25th. | Br. | 1st. | M. | 5½ |
| 10 | Oct. 10th | 130 | Left lower quarter. | Oct. 30th. | Ver. | 1st. | F. | 8 |
| 10 | 1st stage labor | 142 | | | | | | |
| 11 | Oct. 6th | 128 | Right lower quarter. | Nov. 1st. | Ver. | 2d. | F. | 8¼ |
| 11 | Oct. 10th | 128 to 140 | | | | | | |
| 11 | Oct. 27th | 120 | | | | | | |
| 11 | 2nd stage labor | 130 | | | | | | |
| 12 | Oct. 8th | 128 | Left lower quarter. | Nov. 1st. | Ver. | 2d. | M. | 11 |
| 12 | Oct. 22nd | 132 | | | | | | |
| 13 | Oct. 31st | 124 | Median line between pubes and umbilicus. | Oct. 31st. | Ver. | 1st. | M. | 9 |
| 14 | Oct. 28th | 132 | All over abdomen. | Oct. 31st. | Ver. | 1st. | M. | 8 |
| 15 | Nov. 4th | 122 | Left lower quarter. | Nov. 9th. | Ver. | 1st. | F. | 7¼ |
| 15 | 1st stage labor | 144 | | | | | | |
| 16 | Oct. 20th | 134 to 145 | " " | Nov. 4th. | Ver. | 1st. | F. | 7 |
| 17 | Oct. 20th | 118 to 140 | Left lower quarter. | Nov. 9th. | Ver. | 2d. | F. | 8 |
| 17 | Oct. 22nd | 120 | | | | | | |
| 18 | Nov. 10th | 132 | " " | Nov. 11th. | Ver. | 1st. | F. | 6 |
| 19 | Oct. 2nd | 140 | " " | Nov. 13th. | Ver. | 1st. | F. | 7½ |
| 19 | Nov. 6th | 134 | | | | | | |
| 20 | Nov. 2nd | 132 | Right lower quarter. | Nov. 13th. | Ver. | 2d. | F. | 7 |
| 21 | Oct. 28th | 130 | Left lower quarter. | Nov. 13th. | Ver. | 2d. | M. | 7¼ |
| 21 | Nov. 2nd | 144 | | | | | | |
| 22 | Nov. 16th | 130 | " " | Nov. 16th. | Ver. | 1st. | M. | 8½ |
| 23 | Oct. 31st | 122 | " " | Nov. 18th. | Ver. | 2d. | M. | 8 |
| 23 | Nov. 3rd | 132 | | | | | | |
| 24 | Oct. 10th | 130 | Right lower quarter. | Nov. 23rd. | Ver. | 2d. | M. | 7 |
| 24 | Oct. 13th | 140 | Over most all uterine tumor, most intense on right side. | | | | | |
| 24 | Nov. 4th | 128 to 140 | | | | | | |
| 24 | Nov. 15th | 126 to 140 | | | | | | |
| 25 | Oct. 9th | 150 | Left lower quarter. | Nov. 25th. | Ver. | 1st. | M. | 10 |
| 25 | Oct. 13th | 140 | | | | | | |
| 25 | Oct. 22nd | 140 | | | | | | |
| 25 | Nov. 15th | 134 | | | | | | |

| No. of Patient. | Date of Observation. | Pulsation of Heart. | Where Heard. | Date of Delivery. | Presentation. | Position. | Sex. | Weight. lbs. |
|---|---|---|---|---|---|---|---|---|
| 26 | Nov. 10th | 124 | Left lower quarter. | Nov. 27th. | Ver. | 1st. | M. | 8¼ |
| 26 | Nov. 23rd | 118 | | | | | | |
| 26 | Nov. 25th | 124 | | | | | | |
| 27 | Nov. 25th | 150 | " " | Nov. 26th. | Ver. | 1st. | F. | 6 |
| 28 | Oct. 27th | 130 to 180 | Over two-thirds tumor, most intense in right lower quarter. | Nov. 30th. | Ver. | 2d. | M. | 9½ |
| 28 | Nov. 23rd | 128 to 140 | | | | | | |
| 29 | Nov. 30th | 132 | Right lower quarter. | Nov. 30th. | Ver. | 2d. | F. | 7 |
| 29 | 2nd stage labor | 146 | | | | | | |
| 30 | Nov. 10th | 120 | Right lower quarter. | Dec. 3rd. | Ver. | 2d. | M. | 8 |
| 30 | Nov. 15th | 140 | | | | | | |
| 30 | Nov. 23rd | 140 | | | | | | |
| 31 | Nov. 2nd | 152 | " " | Dec 5th. | Ver. | 2d. | M. | 9 |
| 31 | Nov. 15th | 140 | | | | | | |
| 31 | Nov. 23rd | 148 | | | | | | |
| 32 | Oct. 31st | 140 | Left lower quarter. | Dec 8th. | Ver. | 1st. | M. | 8 |
| 32 | Nov. 18th | 140 | | | | | | |
| 33 | Nov. 8th | 124 | " " | Dec. 23rd. | Ver. | 1st. | M. | 12 |
| 33 | Nov. 15th | 128 to 160 | | | | | | |
| 33 | Nov. 23rd | 132 | | | | | | |
| 34 | Nov. 26th | 140 | " " | Dec. 25th. | Ver. | 1st. | F. | 6 |
| 34 | Dec. 1st | 124 to 130 | | | | | | |
| 35 | Dec. 28th | 140 to 160 | " " | Dec. 31st. | Ver. | 1st. | F. | 7 |
| 35 | Dec. 30th | 130 | | | | | | |
| 36 | Dec. 26th | 130 | " " | Dec. 30th. | Ver. | 1st. | F. | 9 |
| 37 | Dec. 31st | 150 to 180 | Right lower quarter. | Jan. 3rd. | Ver. | 2d. | M. | 8 |
| 38 | Dec. 26th | 134 | All over tumor. | Jan. 11th. | Ver. | 1st. | F. | 8¼ |
| 39 | Dec. 28th | 130 | Left lower quarter. | Jan. 7th. | Ver. | 1st. | F. | 7 |
| 39 | Dec. 31st | 138 | | | | | | |
| 40 | Dec. 5th | 132 | Right lower quarter. | Jan. 9th. | Ver. | 2d. | F. | 8½ |
| 40 | Dec. 11th | 130 | | | | | | |
| 41 | Dec. 11th | 132 | Left lower quarter. | Jan. 1st. | Ver. | 1st. | F. | 7 |
| 42 | Jan. 11th, 1874 | 126 | Right lower quarter. | Jan. 12th. | Ver. | 2d. | F. | 7 |
| 43 | Dec. 26th | 140 | Left lower quarter. | Jan. 13th. | Ver. | 1st. | F. | 8 |
| 43 | Dec. 31st | 164 | | | | | | |
| 44 | Jan. 27th, 1874 | 170 | " " | Jan. 28th. | Ver. | 1st. | F. | 6¼ |
| 45 | Nov. 23rd | 122 to 180 | Right lower quarter. | Jan. 16th. | Ver. | 2d. | F. | 11½ |
| 45 | Dec. 11th | 128 | | | | | | |
| 46 | Feb. 8th, 1874 | 128 | " " | Feb. 8th. | Ver. | 1st. | M. | |
| 47 | Jan. 8th, 1874 | 150 | Left lower quarter. | Feb. 9th. | Ver. | 1st | F. | |
| 48 | Jan. 16th | 164 | " " | Feb. 13th. | Ver. | 1st. | M. | |
| 49 | Jan. 3rd | 130 | " " | Feb. 14th. | Ver. | 1st. | M. | |
| 50 | Jan. 12th | 130 | Right lower quarter. | Feb. 15th. | Ver. | 1st. | M. | |

There were 88 examinations made upon these 50 cases, of which there were 23 boys and 27 girls. The lowest pulse was 118; it occurred only twice—once each in the case of a girl and boy. The highest was 180, occurring three times, two of the cases being boys, the other a girl. The average pulse of the males was

136.3; of the females, 137; of both sexes, 136.7. At 136, then, as the dividing line between the sexes—the pulse of this number and below being considered as belonging to boys, and above it to girls—the diagnosis was correct in 26 cases and wrong in 24. Again, leaving out of consideration those examinations where the fœtus was found to be active, the average pulse of the males was 133.6; of the females, 136.2; of both sexes, 134.9. Then, with 134 as the dividing line, the diagnosis was correct in 24 cases and wrong in 22. Again, with 128 as the dividing line, the diagnosis was correct in 28 cases and wrong in 22. There were six girls whose pulse was steady below 128. Five boys had a steady pulse between 128 and 138; three between 138 and 148; two between 148 and 158; and one between 158 and 168. So far as the facts elicited from the entire number of cases are of any value, it is too evident that they have utterly failed to determine the sex in utero. Dr. W. admits the necessity of repeated examination in each case, but says many of his observations were taken but once. Possibly if we had availed ourselves of this repeated examination to a greater extent the result might have been different.

In regard to diagnosing the presentation, we can report more favorably, being correct in 49 out of the 50 cases. The case of failure was one where the breech was the presenting part, but the examination was not made till the second stage of labor. The position, so far as being to the right or left of the median line is concerned, was correct in 38 and wrong in 8 cases. Of the four remaining cases, the heart-sounds were heard all over the uterine tumor in two of them, both being vertex presentations, with the occiput to the left; in the other two cases, the heart-sounds were the plainest on the median line between the pubes and umbilicus, one being a vertex presentation, with the occiput to the left, and the other a breech.

In conclusion, it would seem that an opinion in regard to the sex of the child, founded on the action of the fœtal pulse, is of little more value than a guess, while the presentation may accurately be diagnosed, as possibly likewise the exact position.

312 West Indiana Street.

